# Coding the negative emotions of family members and patients among the high‐risk preoperative conversations with the Chinese version of VR‐CoDES

**DOI:** 10.1111/hex.13502

**Published:** 2022-04-21

**Authors:** Liru Qian, Xinchun Liu, Meng Yin, Ya Zhao, Bingyu Tie, Qingyan Wang, Yi Zhang, Siyang Yuan

**Affiliations:** ^1^ Department of Clinical Psychology, Third Xiangya Hospital Central South University Changsha Hunan China; ^2^ Department of International Exchange and Cooperation, Third Xiangya Hospital Central South University Changsha Hunan China; ^3^ Department of Medical Administration, Third Xiangya Hospital Central South University Changsha Hunan China; ^4^ Dental Health Services Research Unit University of Dundee Dundee UK

**Keywords:** conversation themes, high‐risk preoperative conversation, negative emotions, Verona Coding Definitions of Emotional Sequences, VR‐CoDES

## Abstract

**Background:**

Little is known about family members' and patients' expression of negative emotions among high‐risk preoperative conversations.

**Objectives:**

This study aimed to identify the occurrence and patterns of the negative emotions of family members and patients in preoperative conversations, to investigate the conversation themes and to explore the correlation between the negative emotions and the conversation themes.

**Methods:**

A retrospective study was conducted using the Chinese version of Verona Coding Definitions of Emotional Sequences (VR‐CoDES‐C) to code 297 conversations on high‐risk procedures. Inductive content analysis was used to analyse the topics in which negative emotions nested. The *χ* ^2^ Test was used to test the association between the cues and the conversation themes.

**Results:**

The occurrence rate of family members' and patients' negative emotions was very high (85.9%), much higher when compared to most conversations under other medical settings. The negative emotions were mainly expressed by cues (96.4%), and cue‐b (67.4%) was the most frequent category. Cues and concerns were mostly elicited by family members and patients (71.6%). Negative emotions were observed among seven themes, in which ‘Psychological stress relating to illness severity, family's care and financial burden’ (30.3%) ranked the top. Cue‐b, cue‐c and cue‐d had a significant correlation (*p* < .001) with certain themes.

**Conclusions:**

Family members and patients conveyed significantly more negative emotions in the high‐risk preoperative conversations than in other medical communications. Certain categories of cues were induced by specific emotional conversation contents.

**Patient Contribution:**

Family members and patients contributed to data.

## 1 INTRODUCTION

Misery, disgust, anger, anxiety, fear, sadness and guilt are identified as basic negative emotions.^1^ Negative emotions are common in patients^2^, ^3^ and family members when providing care for the patients.^4^, ^5^ Bo et al.^6^ found that anxiety and depressive emotions had a detection rate of 11.00% and 28.67%, respectively, in hospitalized patients. A meta‐analysis showed that a high prevalence of psychological distress was found among family members^7^, and different studies indicated that patients and their family members present negative feelings verbally or nonverbally during doctor–patient communication.^8^, ^9^ However, negative emotions are not easy to identify.^10^ The Verona Coding Definitions of Emotional Sequences (VR‐CoDES), a coding system that was developed to analyse emotional communication in provider–patient encounters,^11^ defines concerns as an explicit and clear verbalization of an unpleasant emotional state and cues as a verbal or nonverbal hint to an underlying unpleasant emotion.^12^ Patients' hopes, uncertainties, feelings and concerns are expressed indirectly in various medical interviews.^10^ Expressing emotional cues and concerns has certain medical implications, such as allowing patients to put their emotions into words and thereby equipping the doctors to recognize patients' psychological distress.^13^ However, physicians tend to miss most cues and concerns along with adopting behaviours that discourage any disclosure.^14^ While patients are implicit in their expression of concerns,^15^ doctors' interactions with patients are mainly focused on obtaining information useful for diagnosis and treatment, which results in limited exploration of worries or issues relating to the condition of the patients.^16^, ^17^ Additionally, family members might be unable to fully process the information if their emotional distress has not been addressed.^18^

As negative emotions are correlated with insomnia,^19^ poor interpersonal relationship,^20^ poor judgement^21^ and extreme behaviours,^22^ we believe that it is very important to identify negative emotions and explore an appropriate coping strategy in the context of poor doctor–patient relationships in China. Patients and their family members, who have inadequate medical knowledge, might experience more negative emotions when confronting decisions on critical health issues such as cancer diagnosis, treatment options and major operations with high risks. According to a consensus among Chinese hospitals, we define high‐risk procedures as operations, treatments or examinations with high possibility of unexpected negative outcomes during or after the procedures, which are often linked to high medical costs and may result in serious complications, unsatisfactory therapeutic effects and higher death rates. In such circumstances, patients and their family members are stressed and worried due to the pressure of huge uncertainty and critical condition of the patient. Thus, issues raised in high‐risk preoperative conversations and the effectiveness of the communication are important. The preoperative conversation is a means to provide medical information and also acts as a link to strengthen the connection between doctors and patients. However, addressing the complexity of emotional communication is not an easy task.^11^ Reliable and validated scales or coding systems are helpful to identify the emotional expressions and evaluate the responding patterns in the conversation. The VR‐CoDES, a coding system that was developed to analyse these kinds of emotional expressions, has been widely applied in many medical settings that involve oncology, general internal medicine, dentistry, paediatrics, psychiatry and general practice.^8^, ^11^, ^13^, ^23^, ^24^ Nevertheless, little is known about the negative emotions in preoperative conversations regarding high‐risk procedures, and how emotional cues are related to the conversation themes has rarely been explored.

To understand the negative emotional patterns under the high‐risk preoperative conversation setting and to fill the evidence gap, the present study aimed to (1) code the emotional expressions of the family members and patients by using the Chinese version of VR‐CoDES^25^; (2) identify the emotional themes of the conversations; and (3) explore the association between the negative emotions and the themes of the conversations.

## 2 METHODS

### 2.1 Design, sample and setting

We performed a retrospective study to investigate emotional expressions in the high‐risk preoperative procedures. The study was conducted in a tertiary general hospital in Hunan, China. The high‐risk procedures in our study mainly included Grade 3 and Grade 4 operations (Grade 1–4 from easy to difficult, stipulated by the Chinese Government Health Administration). Operations adopted brand new technology, organ transplantation, limb or organ removal and so forth. Operations involving special populations such as elderly patients, pregnant woman with complicated conditions and severely ill newborns were also included. The major characteristics of the procedures were critical or complex condition, long duration of illness and presence of two or more comorbidities. The conversations were mainly about (1) patients' current condition and prognosis; (2) medical measures to be taken and the potential risks of these measures; (3) alternative treatment options; and (4) other issues that need family members' and patients' understanding and cooperation. The majority of the family members were patients' spouses, parents or adult children. Patients' siblings and extended family members were included in the conversations, but on rare occasions.

### 2.2 Procedure

The high‐risk preoperative conversations occurred at the meeting room of the Department of Medical Service in the Third Xiangya Hospital of Central South University between January 2017 and April 2019. A digital audio‐recorder was applied to audiotape the conversations, and participants' approvals of recording were obtained at the start. We randomly collected 300 audio records from the database. Of these 300 audiotapes, 3 were excluded due to poor recording quality, and 297 conversations were included and analysed in our study. Authors had access to information that could identify individual participants during or after data collection. Two coders, Meng Yin (M. Y.) and Ya Zhao (Y. Z.), who had been trained and qualified to use the coding manual of the Chinese version of VR‐CoDES (VR‐CoDES‐C), coded the conversations. One conversation was divided into several ‘sequences’ or ‘turns’ by the coders, and then they coded the sequences that started with the first mention of emotional cues or concerns and ended coding when the topic shifted.^12^ The coding procedure followed four steps. Step 1, 12 conversations were selected randomly and coded independently by two coders, and then disagreements were discussed to reach consensus. Step 2, 30 conversations were selected randomly and coded independently by the two coders to establish inter‐rater reliability. To avoid fatigue and learning effects in the coding procedure, the coders only coded six conversations per day, and all codes were completed in 5 days. Step 3, M. Y. coded the 30 conversations again 2 weeks later to establish intra‐rater reliability. Step 4, after reliability was established, M. Y. coded the rest of the conversations within 1 month.

### 2.3 Analysis

The interaction analysis was conducted using VR‐CoDES‐C to identify patients' emotional expressions of cues and concerns. Based on the original VR‐CoDES, the Chinese version closely follows the definition of cue and concern, as well as the category division. Cues were divided into seven subcategories (Table 1). The intraclass correlation coefficient (ICC) was recommended as a reliable measure to examine the inter/intra‐rater reliability according to previous empirical research when coding doctor–patient interactions using similar schemes to VR‐CoDES.^26^ The reliability of the VR‐CoDES‐C (ICC = .79) was acceptable, and its validity (specificity = 0.99, sensitivity = 0.96) was good.^25^ The inductive content analysis^27^ was performed to explore the emotional contents of identified cues/concerns. This analysis included three main phases: preparation, organizing and reporting. In the preparation phase, researchers input the recognized cues/concerns into N‐Vivo and made a preliminary interpretation of these emotional contents. In the organizing phase, researchers refined these preliminary interpretations which containing cues/concerns by using descriptive words or a shorter phrase. In this phase, these descriptive words were grouped under higher‐order headings. In the reporting phase, these condensed descriptors were then grouped into the overarching final themes. Qualitative data were analysed by N‐Vivo 12.0.

**Table 1:** Definitions of subcategories of cues and concerns with examples in this study

Expression	Definition	Example in high‐risk preoperative conversation
Concern	A clear and unambiguous expression of an unpleasant current or recent emotion that is explicitly verbalized with or without a stated issue of importance	Patient: I told him I'm worried about this problem.
Cue‐a	Words or phrases in which the patient uses vague or unspecified words to describe his/her emotions	Patient: I have to resign myself to this misfortune.
Cue‐b	Verbal hints to hidden concerns (emphasizing, unusual words, unusual description of clinical signs, profanities, exclamations, metaphors, ambiguous words, double negatives, expressions of uncertainties and hope)	Family member: It is only an ordinary operation to doctors, but it is an earth‐shaking event for us.
Cue‐c	Words or phrases that emphasize (verbally or nonverbally) physiological or cognitive correlates (regarding sleep, appetite, physical energy, for example) of unpleasant emotional states.	Family member: I could not sleep in the past few days for thinking about his illness.
Cue‐d	Neutral expressions that mention issues of potential emotional importance that stand out from the narrative background and refer to stressful life events and conditions	Patient: The distance between home and hospital is too far.
Cue‐e	A patient‐elicited repetition of a previous neutral expression (repetitions, reverberations or echo of a neutral expression within a same turn are not included)	Patient: I think it's safer to be hospitalized.
Cue‐f	Nonverbal expressions of emotion	Patient: (sigh) OK, fine…
Cue‐g	Clear expression of an unpleasant emotion, which occurred in the past (more than 1 month ago) or is without a time frame	Family member: We all hope that he will get better, but he said he wanted to give up his life for some time.

The statistical analysis was conducted and descriptive statistics were reported using SPSS 24.0 (IBM Corp.). *χ* ^2^ Was used to test a possible association between cue‐b, cue‐c, cue‐d and the identified themes, and Cramer's *V* was used to indicate the degree of correlation. *p* Values less than .05 were considered statistically significant.

### 2.4 Ethical consideration

Written informed consents were obtained at the start of the conversations from the participating family members, patients and doctors, and the consents indicated their agreement on audio recording and the usage of the audiotapes for further analysis. The collection of the 297 audio records for this study was approved by the Ethics Committee of the Third Xiangya Hospital of Central South University.

## 3 RESULTS

### 3.1 Sample characteristics

Four hundred and thirty family members and 109 doctors participated in the conversations. As nearly half of the documents did not indicate the gender of the family members, this information was absent. Two hundred and ninety‐two patients (male = 154, female = 138) were involved in this study, but only 39 patients participated in the conversations by themselves. Other patients did not participate because they were critically ill and unable to be part of the conversation, or patients were newborn or young children, or family members decided not to share the full details of the illness with the patients. The distribution of conversations in clinical departments is shown in Table 2.

**Table 2:** The distribution of conversations in clinical departments

Department	Number	Percentage (%)
Organ transplant	34	11.6
Urinary surgery	31	10.6
General surgery	29	9.9
Orthopaedics	27	9.2
Gynaecology	27	9.2
Otolaryngology	21	7.2
Liver and pancreas surgery	18	6.2
Ophthalmology	17	5.8
Cardiothoracic surgery	17	5.8
Obstetrics	13	4.5
Gastrointestinal surgery	9	3.1
Haematology	7	2.4
Intensive care unit	5	1.7
Neurosurgery	5	1.7
Breast and thyroid surgery	5	1.7
Oncology	4	1.4
Gastroenterology	4	1.4
Neurology	3	1.0
Endocrinology	3	1.0
Respiratory	3	1.0
Radiology	3	1.0
Emergency	2	0.7
Traditional Chinese Medicine	1	0.3
Plastic surgery	1	0.3
Nephrology	1	0.3
Geriatrics	1	0.3
Paediatrics	1	0.3
Total	297	100.0

### 3.2 Inter‐rater reliability and intra‐rater reliability

The ICCs for inter‐rater reliability were .87, .76 and .71 on the identification of cues/concerns, subcategories of cues and the initiation of cues and concerns, respectively. The ICCs for intra‐rater reliability were .88, .83 and .74 on the identification of cues/concerns, subcategories of cues and the initiation of cues and concerns, respectively.

### 3.3 The duration and frequency of expressions

A total of 255 (85.9%) out of 297 recordings were coded with at least one cue or concern. The average conversation length was 27.09 min, ranging from 7.53 min to 1.17 h. A total of 1483 cues/concerns were identified, with a mean of 4.99 cues or concerns per conversation. No cue or concern was identified for the remaining 42 conversations.

### 3.4 Patients' emotional expressions

#### 3.4.1 Expressions of cues/concerns

Cues and concerns, respectively, accounted for 96.4% (*n* = 1430) and 3.6% (*n* = 53) of the total conversations. A total of 1430 cues were coded into seven subcategories. The frequencies of different categories of cues and concerns are shown in Table 3. The most common category of cues was cue‐b, which accounted for 67.4% (*n* = 964). Cue‐d (*n* = 272, 19%) was the second most common category. The remaining subcategories included cue‐c (*n* = 90, 6.3%), cue‐f (*n* = 67, 4.7%), cue‐e (*n* = 30, 2.1%), cue‐g (*n* = 6, 0.4%) and cue‐a (*n* = 1, 0.1%).

**Table 3:** Frequency and percentage of cues and concerns

Expression	Frequency, *n* (%)	PE, *n* (%)	HPE, *n* (%)
Cue‐a	1 (0.1)	0 (0)	1 (0.2)
Cue‐b	964 (67.4)	640 (60.3)	324 (77.0)
Cue‐c	90 (6.3)	76 (7.2)	14 (3.3)
Cue‐d	272 (19.0)	252 (23.7)	20 (4.8)
Cue‐e	30 (2.1)	29 (2.7)	1 (0.2)
Cue‐f	67 (4.7)	19 (1.8)	48 (11.4)
Cue‐g	6 (0.4)	6 (0.6)	0 (0)
Concern	53 (3.6)	40 (3.7)	13 (3.1)
Total	1483 (100.0)	1062 (100.0)	421 (100.0)

Abbreviations: HPE, health‐provider‐elicited; PE, patient/family member‐elicited.

Among the 1483 cues/concerns, 71.6% were elicited by family members and patients (PE, *n* = 1062) and 28.4% were elicited by doctors (HPE, *n* = 421). The result indicated that the all cues/concerns, except for cue‐f, were largely initiated by family members and patients, but cue‐f was largely elicited by doctors. The source of subcategories of cues is presented in Table 3.

#### 3.4.2 Themes of emotional contents

**Figure 1:**
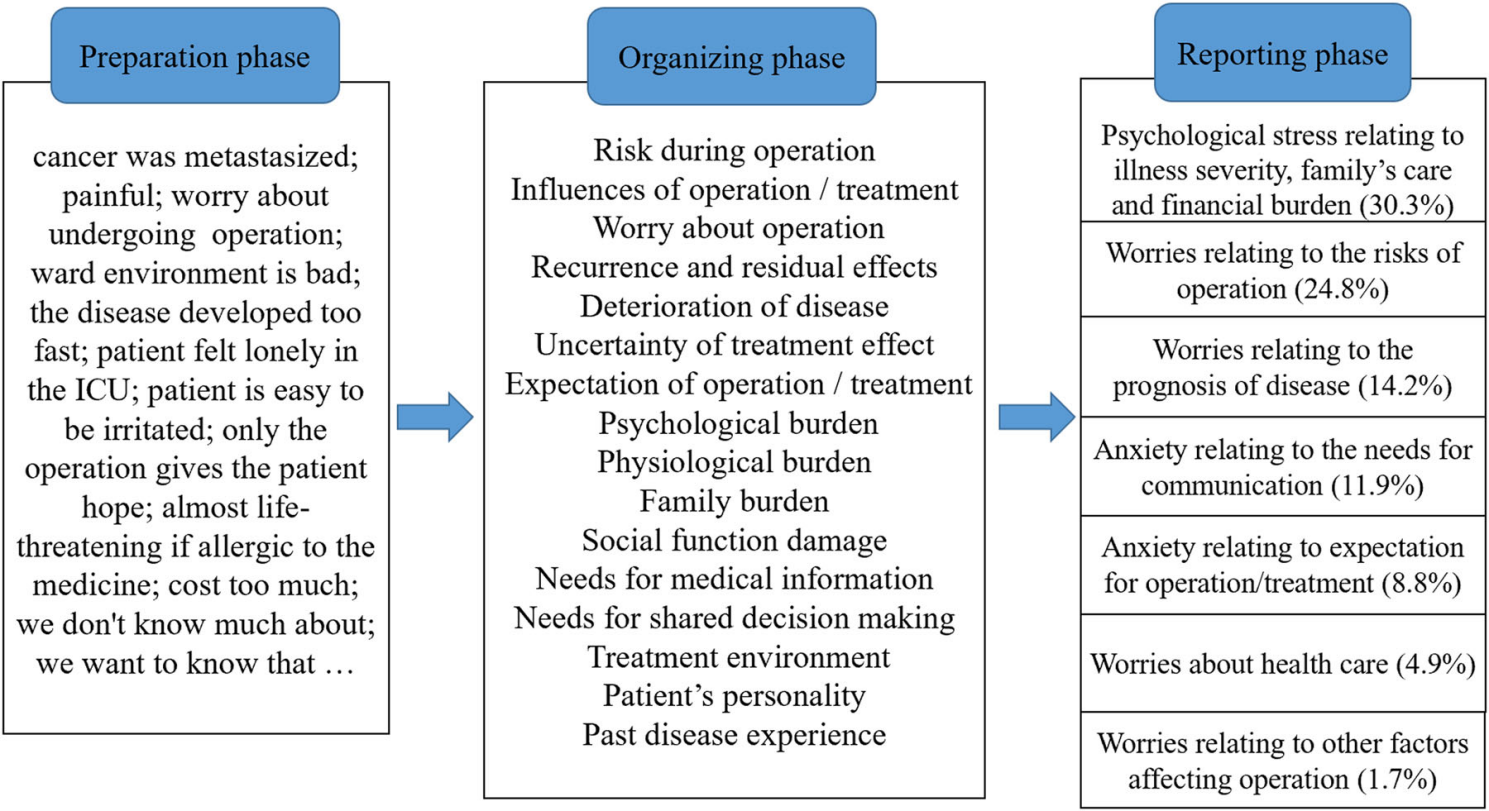
The process of content analysis

After deleting invalid content (3.4%), content analysis resulted in seven overall themes (Figure 1), namely, (1) Psychological stress relating to illness severity, family's care and financial burden, (2) Worries relating to the risks of operation, (3) Worries relating to the prognosis of disease, (4) Anxiety relating to the needs for communication, (5) Anxiety relating to expectation for the operation/treatment, (6) Worries about healthcare and (7) Worries relating to other factors affecting the operation.

The most common theme was ‘Psychological stress relating to illness severity, family's care and financial burden,’ which accounted for 30.3% among the total themes. The largest proportion of these burdens was the feelings of helpless or despair. For instance, some of the family members said ‘I'm afraid that even the doctors may have no solutions for his (the patient's) severe condition’. They were conveyed both verbally (e.g., patient: ‘I feel stressful, so I'm afraid of the operation’) and nonverbally, (e.g., cry, sniff and sigh). Family burden was the second most common content, of which financial burden was the major concern. High‐risk operations are always very complicated and always demand long‐term healthcare, which means a huge care workload and medical expenditures for a family. As family members voiced ‘We borrowed money from relatives because we have no income’, or ‘We have to work, can not take good care for her recovery by ourselves’.

The theme ‘Worries relating to the risks of operation’ (24.8%) ranked the second, including the risks during the operation, the impact of the operation, concerns about the failure of the operation and anaesthesia risks. Most family members and patients worried about the huge risks that they had to bear during the operation (e.g., patient: ‘Anyway, I need to take a great deal of risks in this operation’), and they usually sighed to express their concerns. Many family members and patients worried that patients' poor condition could not endure the operations (e.g., patient: ‘I'm afraid that my liver can't stand’, or ‘He is too weak and has high blood pressure, I am worried that he will not be able to bear the operation’).

The third most common theme was ‘Worries relating to the prognosis of disease’ (14.2%), which was related to disease progression, deterioration after operation or treatment. Some family members were concerned whether the patient's conditions would improve or deteriorate. They usually expressed their concerns by asking their doctors about this (e.g., ‘Will my daughter get worse in the future?’). Other contents referred to complications, recurrence and uncertainty of treatment effects. Worries concerning complications were identified mostly among conversations related to eye operations. Many family members worried if the patient's vision would be affected after the operation. In terms of uncertainty of treatment effects, family members and patients were mainly concerned about whether the patients' physical condition would recover or the operation could completely resolve the issue (e.g., ‘After the operation, will these symptoms disappear?’).

The remaining contents included ‘Anxiety relating to the needs for communication’ (11.9%), ‘Anxiety relating to expectation for operation/treatment’ (8.8%), ‘Worries about healthcare’ (4.9%) and ‘Worries relating to other factors affecting operation’ (1.7%). Needs for communication mainly included disease/treatment‐related information and shared decision‐making. Family members often cared about the operation approaches and felt puzzled about making decisions (e.g., ‘When making a decision, I feel entangled’). Expectation for operation/treatment was mainly about hope. They desperately asked physicians to take the patients' illness seriously and hoped that patients could receive the best treatment (e.g., patient: ‘Please do everything you can to help me’). Some family members wanted independent wards to provide patients with a better environment for treatment and recovery. Some family members and patients were concerned about the diet before and after the operation. Factors affecting the operation mainly included patients' personality and illness history. Some family members worried that patients' personality characteristics and history of previous diseases would have an influence the outcomes of the operation and postoperative recovery (e.g., ‘She is short‐tempered’).

### 3.5 Correlation between cues and themes

When talking about different themes, family members and patients tended to use different cues to express negative emotions (*p* < .001, *χ* ^2^ = 125.544, *DF* = 12). A slight but significant correlation was presented between cue‐b, cue‐c, cue‐d and the themes (*p* < .001, Cramer's *V* = 0.221). The distribution of the subcategories and the themes is listed in the cross‐tabulation below (Table 4). Our study found that cue‐b was the most common expression across all themes, except for ‘Worries relating to other factors affecting operation’, and it was mostly presented in the two themes of ‘Worries relating to the risks of operation’ and ‘Psychological stress relating to illness severity, family's care and financial burden’. Cue‐c and cue‐d were mostly presented in the theme of ‘Psychological stress relating to illness severity, family's care and financial burden’. Cue‐d was presented in the themes of ‘Worries relating to the prognosis of the illness’ and ‘Anxiety relating to the needs for communication’ with a relatively high frequency.

**Table 4:** The distribution of the subcategories and the themes

Cue	Psychological stress relating to illness severity, family's care and financial burden	Worries relating to the risks of operation	Worries relating to the prognosis of the disease	Anxiety relating to the needs for communication	Anxiety relating to expectation of operation/treatment	Worries about healthcare	Worries relating to other factors affecting the operation
Cue‐b	251 (27.1%)	255 (27.6%)	150 (16.2%)	115 (12.4%)	107 (11.6%)	37 (4.0%)	10 (1.1%)
Cue‐c	54 (62.8%)	8 (9.3%)	1 (1.2%)	8 (9.3%)	12 (14.0%)	2 (2.3%)	1 (1.2%)
Cue‐d	90 (33.5%)	38 (14.1%)	45 (16.7%)	46 (17.1%)	8 (3.0%)	30 (11.2%)	12 (4.5%)

## 4 DISCUSSION

Anxieties and worries are common among family members and patients when they have preoperative conversations with the doctors, especially when the operations are accompanied by a high risk of potential failure or unexpected outcomes. Very few studies had previously been conducted to identify the negative emotional expressions among high‐risk preoperative conversations. We performed an investigation on these kinds of conversations by using the Chinese version of VR‐CoDES to identify the negative emotions. We hypothesized that the occurrence rate of negative emotional disclosure would likely be higher than ordinary medical consultations, and there might be some correlation between the VR‐CoDE's coding of negative emotions and the conversation topics. In this study, we found that the occurrence rate of negative emotions was very high (85.9%) during the whole process of conversations, and it was higher than in many previous studies, in which the occurrence rates ranged from 50% to 76%.^28^, ^29^, ^30^ The average number of cues or concerns per conversation in this study was also higher than that in other studies, whose mean ranged from 1.6 to 4.0,^28^, ^30^, ^31^ except for those talks conducted for children^23^ or when conveying bad news to cancer patients.^32^ We also found that cue‐b, c and d were closely related to certain conversation themes, among which ‘Worries relating to illness severity, family's care and financial burden’ and ‘Worries relating to the risks of operation’ were the most common ones. This study provided additional evidence to the application of VR‐CoDES, and initially explored negative emotions among preoperative conversations by using this coding tool. In this study, we observed a very high occurrence of negative emotional cues and concerns among the talks. This high frequency of negative emotions can be mainly explained by the psychosocial burdens that the high‐risk procedures placed on both family members and patients. The average duration of these conversations was nearly half an hour, which is obviously longer than daily healthcare communication in the Chinese context, which is always less than 15 min,^9^ and might also be the potential reason for identification of more cues and concerns as there were more opportunities for family members and patients to express emotions. Family members and patients expressed more cues (96.4%) than concerns (3.6%), and this finding suggested that their hidden worry was conveyed in an indirect way. Interestingly, this phenomenon can be partly linked to the Chinese culture. As China is deeply influenced by the ideology of Confucianism and collectivism, people tend to express emotions in an indirect way and are in awe of authority. Thus, when family members and patients regard doctors as authority figures, they tend to behave in a cautious way and convey their emotions in an indirect way. A previous study found^33^ that doctors as experts are in charge of communication to provide medical information and patients accept advice from clinicians passively and voice their concerns with tentative narratives, which leads to limited emotional expression by patients. This means that healthcare providers should pay more attention to patients' implicit expressions, which are likely to be accompanied by a certain degree of negative emotions.

We also observed that the most frequent category of cues identified in this study was cue‐b, the verbal hints to hidden concerns such as emphasizing, unusual words, expressions of uncertainties and hope. Even though we know that this result is similar to that reported by many other studiesh,^9^, ^28^, ^32^, ^34^ we are still impressed by the intensity of cue‐b taking into account of the content of the conversation. The high level of anxiety was obviously conveyed by anxious queries, emphasizing and repeated expressions of hope. Cue‐a, words or phrases in which the patient uses vague or unspecified words to describe his/her emotions, was only presented in this study once (e.g., patient: ‘I have to resign myself to this misfortune’). This may be because the doctors mainly focus on the condition rather than asking questions about the patient's emotional experience. Therefore, family members and patients prefer to convey emotions through consultation about the operation or treatment, or directly expressed their concerns, instead of using words or phrases to describe emotions ambiguously. Limited studies have reported that cue‐f, nonverbal cues such as crying and sighing, was mainly elicited by healthcare providers. However, in our study, these nonverbal cues were often expressed by patients or family members after the doctor stated the risks of the operation.

The negative emotions, namely, here, cues and concerns, were mainly expressed by family members and patients. In the present study, family members and patients actively expressed their needs and hopes. One reason may be that family members and patients with a high level of negative emotions needed to take many factors into consideration in such high‐risk preoperative conversations.

We observed certain themes among the conversations. ‘Psychological stress relating to illness severity, family's care and financial burden of family’ (30.3%) was the most common theme that indicated patients'/family members' negative emotions. Psychosocial burden, such as lacking personnel and time to take care of the patients and the high medical cost, can easily cause stress. Similar to an intensive care unit (ICU) study,^35^ family members expressed their worries and concern about the financial burden. This may be because ICU patients also face higher risks, despite the same severity of disease and financial pressure. This indicated that when patients are faced with situations similar to high‐risk operations, medical staff should concentrate on the patients' and their families' burden. The theme, ‘Worries relating to the risks of operation’ (24.8%), was generally discussed before a major operation, but rarely mentioned in general operations.^36^ This may be due to the particularities of high‐risk operations. Doctors need to communicate the risk of the operation with patients and their family members.

In accordance with previous studies, ‘Worries relating to the prognosis of disease’ (14.2%) was mentioned usually in the conversations.^37^, ^38^, ^39^ Family members and patients often talked about the prognosis of the disease with clinicians before the operation, and asked doctors about the outcomes of the treatment, the possibility of recurrence and the complications after the operation.^40^, ^41^ In addition, ‘Anxiety relating to the needs for communication’ (11.9%) was also reported in other preoperative studies.^42^, ^43^ Family members and patients expressed their requests to communicate with healthcare providers. It mainly included the need for disease/therapy‐related information and shared decision‐making as family members and patients do not have adequate professional medical knowledge.

While the themes of ‘Anxiety relating to expectation for operation/treatment’ (8.8%), ‘Worries about healthcare’ (4.9%) and ‘Worries relating to other factors affecting operation’ (1.7%) were mentioned less, these expressions should also be taken notice. In other studies, patients and their family members voiced their expectations about the operation^41^, ^44^ and wanted to know how to take care of patients.^45^ Although ‘Worries relating to other factors affecting operation’ has rarely been discussed in other preoperative research, a study indicated that the patient's personality affected preoperative anxiety.^46^ Therefore, it is recommended that medical staff provide patients with personalized care, given their personalities and individual needs. The identified themes may help medical staff to recognize and deal with the negative emotions of family members and patients.

In terms of the correlation between cues and themes, we obtained some interesting results. More than half of cue‐b was identified among two major themes, namely, ‘Worries relating to illness severity, family's care and financial burden’ and ‘Worries relating to the risks of operation’. This suggests that when talking about topics related to operation risks, doctors should be aware if patients or family members use emphasis, unusual or ambiguous words to express their worries and hope. When such expressions exist in the interactions, family members and patients might have emotional distress. Doctors should respond to these expressions to alleviate the anxiety of family members and patients. Cue‐c mainly occurred in the theme of ‘Worries relating to illness severity, family's care and financial burden’. Cue‐c focuses on physiological or cognitive correlates of unpleasant emotional states. It was not surprising that the majority of cue‐c was found in this theme because when faced with major worries and financial pressures, people's sleep and/or appetite tend to be greatly affected. It is noteworthy that despite the theme of ‘Worries relating to illness severity, family's care and financial burden’, cue‐d was also identified in the themes of ‘Worries relating to the prognosis of the illness’ and ‘Anxiety relating to the needs for communication’. These results demonstrate that family members and patients presented neutral expressions when they talked about the prognosis and conveyed their demands for more information. This implies hidden worries related to these issues.

In summary, we found that more negative emotions may be conveyed during preoperative conversations, especially when those operations or procedures involve high levels of risk. Certain themes of the conversations are found to be associated with cue‐b, cue‐c and cue‐d. The results of our study provided additional evidence to the research of negative emotions in the high‐risk preoperative conversations. These results demonstrated obvious emotional revealing among this kind of communication setting. Therefore, in order to achieve a good shared decision‐making, great attention and proper communicating strategy are needed under a critical medical situation. Future studies will be needed on identification of negative emotions and healthcare providers' response to further examine the correlation between these emotions and certain topics, and to explore the adequate response.

There are several limitations in our study. First, as this was a retrospective study based on data from years ago, it was not possible to verify the coding outcomes with family members and patients who could not be contacted. Second, emotional expressions of family members and patients may be different, but our study did not conduct an analysis of the possible differences because we were unable to identify the individuals due to the limited paper records of the participants. Third, we did not analyse the social demographic characteristics of the participants for the same reason mentioned above.

## 5 CONCLUSION

In conclusion, we identified a high occurrence of negative emotions in the preoperative conversations related to operations deemed high risk, higher than that in most of the usual doctor–patient communication. Family members and patients mainly conveyed their emotions in a vague way, namely, cues, defined by the coding system of VR‐CoDES. Very importantly, we demonstrated that the most frequent cue‐b, cue‐c and cue‐d were correlated with certain themes of the conversation. This suggests that specific topics are more frequently associated with certain emotional expressions.

## AUTHOR CONTRIBUTIONS

Xinchun Liu conceptualized, designed and led the study. Xinchun Liu and Yi Zhang were involved in project administration. Meng Yin, Ya Zhao, Bingyu Tie and Qingyan Wang collected and interpreted the data. Liru Qian, Meng Yin, Ya Zhao and Bingyu Tie analysed the data. Liru Qian wrote the first draft of the manuscript and contributed to revised content for publication. Liru Qian, Xinchun Liu and Siyang Yuan revised the manuscript. All authors approved the final manuscript.

## CONFLICTS OF INTEREST

The authors declare no conflicts of interest.

## Data Availability

Data are available from the corresponding author on reasonable request.
